# Effect of folic acid supplementation on homocysteine concentration and association with training in handball players

**DOI:** 10.1186/1550-2783-10-10

**Published:** 2013-02-21

**Authors:** Jorge Molina-López, José M Molina, Luís J Chirosa, Daniela I Florea, Laura Sáez, Elena Planells

**Affiliations:** 1 Department of Physiology, Institute of Nutrition and Food Technology, University of Granada, Granada 18071, Spain; 2 Department of Physical Education and Sports, Faculty of Sports Sciences, University of Granada, Granada 18071, Spain

**Keywords:** Nutritional status, Sport, Folic acid, Supplementation, Homocysteine

## Abstract

**Background:**

Strenuous physical activity can alter the status of folic acid, a vitamin directly associated with homocysteine (Hcy); alterations in this nutrient are a risk factor for cardiovascular disease. Handball players are a population at risk for nutrient deficiency because of poor dietary habits.

**Objective:**

The aims of this study were to evaluate nutritional status for macronutrients and folic acid in members of a high-performance handball team, and determine the effect of a nutritional intervention with folic acid supplementation and education.

**Design:**

A total of 14 high-performance handball players were monitored by recording training time, training intensity (according to three levels of residual heart rate (RHR): <60%, 60%–80% and >80%), and subjective perceived exertion (RPE) during a 4-month training period. Nutritional, laboratory and physical activity variables were recorded at baseline (Week 0), after 2 months of dietary supplementation with 200 μg folic acid (50% of the recommended daily allowance) (Week 8) and after 2 months without supplementation (Week 16). We compared training load and analyzed changes in plasma concentrations of Hcy before and after the intervention.

**Results:**

Bivariate analysis showed a significant negative correlation (*P* < 0.01) between Hcy and folic acid concentrations (*r* = −0.84) at Week 8, reflecting a significant change in Hcy concentration (*P* < 0.05) as a result of hyperhomocysteinemia following the accumulation of high training loads. At Week 16 we observed a significant negative correlation (*P* < 0.01) between Hcy concentration and training time with an RHR <60%, indicating that aerobic exercise avoided abrupt changes in Hcy and may thus reduce the risk of cardiovascular accidents in high-performance athletes.

**Conclusion:**

Integral monitoring and education are needed for practitioners of handball sports to record their folic acid status, a factor that directly affects Hcy metabolism. Folic acid supplementation may protect athletes against alterations that can lead to cardiovascular events related to exertion during competition.

## Background

Folic acid is a vitamin needed by a number of enzymes essential for DNA synthesis and amino acid metabolism [1]. This nutrient is an important co-factor in the methionine pathway, the most important source of methyl groups in the human organism [2]. Low folic acid intake is known to contribute to increased levels of homocysteine (Hcy) as a result of its interrelation with methionine metabolism [2-6]. Inadequate intake of folic acid has been described in athletes who practice different sports [1], and athletes are often deficient in their intake of total calories, carbohydrate, protein, and micronutrients [7]. Some authors consider supplementation with folic acid as an efficient way to reduce elevated Hcy levels [8,9], and it has been suggested that in certain cases, folic acid supplementation should be used for preventive purposes [10]. Earlier findings have suggested that doses of 0.2 to 0.4 mg/d can achieve maximal reductions in Hcy in healthy young populations, whereas doses up to 0.8 mg/d are needed to reduce Hcy in individuals with coronary heart disease [11].

Regular physical activity (PA) can alter the requirements for some micronutrients [1]. This makes it important to choose foods carefully, taking into account the quality and quantity of macronutrient intakes, since requirements can vary depending on the type of exercise performed [12].

Elevated plasma levels of Hcy are considered a risk factor for cardiovascular disease (CVD) [13]. Regular physical activity is now well established as a key component in the maintenance of good health and disease prevention, and has been specifically recognized to reduce the risk of appearance of CVD by reducing chronic inflammation, which plays a key role in the atherogenic process, blood pressure, body composition, insulin sensitivity and psychological behavior [14,15].

In contrast, acute intense exercise has been shown to increase plasma Hcy concentrations [14]. Several factors have been reported to be associated with increases in Hcy, such as endothelial cell injury, which stimulates vascular smooth muscle cell growth, increases platelet adhesiveness, enhances LDL cholesterol oxidation and deposition in the arterial wall, and directly activates the coagulation cascade [16]. Some research has concluded that Hcy levels may be influenced by the duration, intensity and type of exercise [6,14,17,18], whereas other studies have identified lifestyle factors such as smoking, eating habits and alcohol consumption [6,19,20], as well as age, elevated blood pressure, renal failure [17,21] and genetic factors [22], as factors that contribute to increased plasma concentrations of Hcy. In addition, nutritional factors such as reduced folic acid intake have been implicated [3,13].

Several authors [4,13,22,23] have established a direct relationship between regular physical exercise (PA) and a reduction in CVD risk, although the data regarding the effect of PA on plasma Hcy concentrations remain controversial because of methodological differences among different studies. Murakami et al. [13] noted that these discrepancies may reflect differences in the methods used to evaluate PA, the lack quantitative information on training intensity or training time, and in some cases the lack of adjustment for folate intake status [4]. However, Venta et al. [14] suggested three possible mechanisms that may explain the increase in Hcy with increasing exercise intensity: increased free radical production [15], increases in methylated forms such as creatine and acetylcholine, and increases in the amino acid pool as a result of protein catabolism. The need for research in athletes who take part in different sports has been suggested to be important in order to account for the high prevalence of hyperchromocysteinemia [15]. To date, however, there have been no studies that evaluated plasma Hcy levels while taking into account nutrient intakes, training intensity and training time, and rate of perceived exertion (RPE). Moreover, the relationship between PA and Hcy has not been studied in team sports such as handball, in which intermittent activity alternates with periods of intense aerobic activity [24].

In the present study our aims were to evaluate macronutrient and folic acid nutritional status in high-performance athletes (handball players), and to determine the effect on these parameters of training and a nutritional intervention based on dietary supplementation with folic acid. We analyzed the data in the light of training load and plasma Hcy concentrations.

## Methods

### Participants

The study was done during the February to June 2010 sports season and all participants were members of the handball team (n = 14) sponsored by the Club Deportivo Puente Genil de Balonmano (Granada, Spain), in the Honor B Division of the Spanish professional handball league. The sample comprised 14 men (mean age 22.9 ± 2.7 years) who trained for a mean of 4 days per week in addition to competing in matches on weekends.

Participation in the study was voluntary. None of the participants had evidence of CVD, diabetes or hypertension. All participants provided their informed consent in writing, and were given detailed information at the beginning and end of the study regarding the aims and procedures involved. The study was approved by the Research Ethics Committee of the University of Granada.

### Anthropometric and biochemical measures

Body weight, body mass index and body fat percentage in all participants were determined with a Tanita TBF-300WA Body Composition Analyzer. Height was measured on a scale to within the nearest 0.01 cm.

Blood samples for laboratory analyses were obtained after a 12-h fast after the last training session in each time period. Venous blood was drawn, centrifuged to separate plasma and red blood cells, and stored at −80°C. Folic acid concentration was measured with an electrochemical luminescence immunoassay (ECLIA, Elecsys 2010 and Modular Analytics E 170, Roche Diagnostics, Mannheim, Germany) with a reference value of 3 pg/l [25]. Plasma concentrations of Hcy were measured with a fluorescence polarization immunoassay (IM®, Abbott Laboratories, Abbott Park, IL, USA) [25]. Laboratory values were determined for transferrin, prealbumin, high-density lipoprotein, low-density lipoprotein and total cholesterol to verify adequate nutritional status in all participants and rule out the possibility of nutritional alterations that might have affected the findings.

### Assessment of macronutrient and folic acid intake

To evaluate dietary intakes we used a food consumption questionnaire [26] consistent with a 72-h recall system during 3 consecutive days (2 working days and 1 non-working day). During the educational intervention the participants were instructed to abstain from consuming caffeine or alcohol. Three time points were used during a 4-month period: baseline (Week 0), followed by 2 months of dietary supplementation (Week 8), followed by 2 months without supplementation (Week 16). Food intakes were recorded with the help of a manual containing photographs of standard amounts of different foods and prepared dishes. To record portion sizes and the amounts of different foods as accurately as possible, the participants were asked to identify the foods consumed and describe the size of the portions. Food intakes were analyzed with *Nutriber®* software [27] to convert them into data for absolute nutrient intakes and percentage values of adequate intakes according to individual needs.

Macronutrient intakes (carbohydrates, protein, and fat and folic acid) were compared to reference intakes [28]. Percentage macronutrient intakes referred to total energy intake were compared with recommended dietary allowances (RDA) [29].

### Nutritional supplementation and education intervention

Dietary supplementation consisted of folic acid at 200 μg/d, starting on day 1 in Week 0 and ending on the final day of this 2-month period in Week 8. For the following 2 months no dietary supplementation was used; this period lasted from Week 8 to Week 16, when the study period ended.

The educational intervention was designed ad hoc for this type of study population by a team of nutrition specialists. The intervention consisted of three phases. First, the nutrition team explained aspects related with nutrition in general, with emphasis on the different types of nutrients and their importance for maintaining good health in basically healthy persons. This was followed by education focusing more specifically on nutrition and PA. In this second phase the emphasis was on specific nutritional requirements in persons who perform continuous PA, since nutrition in this population is often not well balanced, and supplements are often used to increase performance [1]. In the third phase, team members responded to the questions participants raised at any time throughout the study period to provide additional information and clarification.

### Training profile

To record training parameters we used three variables that define training load: training time, intensity and RPE. All participants trained for a mean of 4 days per week in addition to participating in competition matches on weekends.

Training time was recorded during a 4-month period covering the professional handball competition season, divided into four 1-month mesocycles. In each training session we recorded the number of minutes spent on each type of exercise until the desired training time was reached. The first 2 months (mesocycles 1 and 2) comprised the period of training when supplementation was used (STp), and the following 2 months (mesocycles 3 and 4) comprised the period of training without dietary intervention (NSTp). Total training time in each mesocycle was calculated as the sum for all training sessions and competition match times.

Training intensity was recorded with Polar S610 and Polar Team pulse meters (Polar Electro Ibérica, Barcelona, Spain) once per training week, for a total of 22 final recorded training sessions (11 for each training period). To calculate maximum heart rate (HR_max_) we used the course navette test of maximum aerobic power. We also recorded baseline heart rate during 7 days to obtain an accurate mean value. Heart rate reserve or residual heart rate (RHR) was calculated as HR_max_ minus basal heart rate to establish the level of intensity and the time each athlete spent in each level [30]. We used three ranges of intensity: <60%, between 60% and 80%, and >80% RHR.

The RPE was used to determine whether the amount of exertion each participant perceived was consistent with actual intensity of exertion once per training week, for a total of 22 final recorded training sessions (11 for each training period). The participants indicated one of the three levels of perceived exertion at the end of each training session. We calculated RPE as the mean ± standard deviation (SD) (*n* = 14) to evaluate perceived load in each mesocycle or month of training.

Training sessions were monitored and standardized by using the same exercises in the same order and with the same duration across sessions. The results were compared as the mean ± SD (*n* = 14) for each of the three study periods.

### Data analysis

The data are reported with descriptive statistics. For numerical variables we used the arithmetic mean, SD and standard error of the mean. The results for categorical variables are reported as percentage frequencies. To determine whether the data fitted a parametric model, the Kolmogorov-Smirnov test was used to verify normal distribution. To check the homoscedasticity of the variables, the Levene test was used. Between-group comparisons were made with the chi-squared test and single-factor analysis of variance. Linear regression analysis was used to identify correlations by calculating Pearson’s bivariate correlation coefficient. All statistical analyses were done with SPSS v. 16.0 for Windows.

## Results

The general characteristics of the participants are shown in Table 1, and these characteristics did not change significantly during any of the three study periods.

**Table 1:** Characteristics of the participants at three time points

**N = 14**						
**Measurement**	**Mean**			**SD**		
Age (years)	22.9			2.7		
Height (m)	1.87			0.06		
	**Week 0**		**Week 8**		**Week 16**	
	**Mean**	**SD**	**Mean**	**SD**	**Mean**	**SD**
Weight (kg)	86.72	5.36	86.47	5.59	86.38	4.81
Body mass index (kg/m^2^)	24.72	1.12	24.61	1.30	24.62	1.14
Body fat (%)	11.58	2.53	11.60	2.45	11.57	2.34

SD, standard deviation.

### Assessment of macronutrient and folic acid intake

Energy, macronutrient and folic acid intakes are summarized in Table 2, and are referred to RDAs for athletes [28,29]. The main finding was a significantly higher (*P* < 0.01) folic acid intake in Week 8 compared to Week 0 and Week 16, as a result of supplementation. When folic acid intake was adjusted for energy intake in Week 8 regardless of supplementation, the difference became nonsignificant.

**Table 2:** Energy, macronutrient and folic acid intakes at three time points

**N = 14**	**RDA**	**Week 0**		**Week 8**		**Week 16**	
		**Mean**	**SD**	**Mean**	**SD**	**Mean**	**SD**
Energy (kcal/kg/day)	44*	34.45	3.56	38.91^a^	4.15	38.54^a^	2.94
Macronutrients (g/day)							
Protein	104 – 147*	133.43	14.32	146.64	35.64	147.04^a^	25.51
Carbohydrate	519 – 865*	360.91	27.64	421.50^a^	49.24	416.80^a^	38.82
Fat	78 – 95*	118.57	22.52	132.22 ^a^	17.75	129.57	21.79
Macronutrients (g/kg/day*)*							
Protein	1.2 - 1.7*	1.54	0.22	1.70	0.44	1.70^a^	0.33
Carbohydrate	6 – 10*	4.17	0.41	4.88^a^	0.60	4.82^a^	0.36
Fat	0.9 – 1.1*	1.37	0.28	1.53^a^	0.19	1.49	0.21
Macronutrients (% energy intake)							
Protein	12 – 15%*	17.97	1.83	17.47	3.73	17.65	2.54
Carbohydrate	45 – 65%*	48.66	4.10	50.21	2.54	50.20	3.62
Fat	20 – 35%*	35.71	4.88	35.51	3.81	34.92	4.01
Vitamins (μg/day)							
Folic acid	400*	301.97	89.05	516.11^a^	54.49	290.35^b^	98.57

RDA, recommended daily allowance. SD, standard deviation.

* Values used for comparison were from previous publications [28,29].

^a^ Statistically significant differences (*P* < 0.05) between Week 0 vs. Week 8 and Week 16.

^b^ Statistically significant differences (*P* < 0.05) between Week 8 vs. Week 16.

Macronutrient intakes were significantly higher (*P* < 0.05) in Week 0 compared to Week 8 and Week 16 for carbohydrates. Fat intake was significantly higher in Week 0 and Week 8, and protein intake was significantly higher in Week 0 and Week 16.

Table 3 shows the percentages of participants whose macronutrient and folic acid intakes were within each tercile of the RDA, or were above the RDA, in each of the three study periods. The results show that folic acid intake was above 100% the RDA in Week 8. In Week 0 and Week 16, intake was below 2/3 of the RDA in 42.9% of the participants [29]. Mean carbohydrate intake was below the RDA [28] at all time points, whereas fat and protein intakes were above 100% of the RDA [28].

**Table 3:** Recommended daily allowance covered for energy, macronutrients and folic acid at three time points

**Nutrient**		**≤ 2/3 RDA**	**> 2/3 RDA ≤ RDA**	**> RDA**
**Macronutrients (%)**				
Protein	Week 0	-	-	100
	Week 8	-	-	100
	Week 16	-	-	100
Carbohydrate	Week 0	35.7	64.3	-
	Week 8	-	92.9	7.1
	Week 16	-	100	-
Fat	Week 0	-	-	100
	Week 8	-	-	100
	Week 16	-	-	100
Vitamins (%)				
Folic acid	Week 0	42.9	42.9	14.3
	Week 8	-	-	100
	Week 16	42.9	50.0	7.1

RDA, recommended daily allowance.

### Training profile

**Figure 1:**
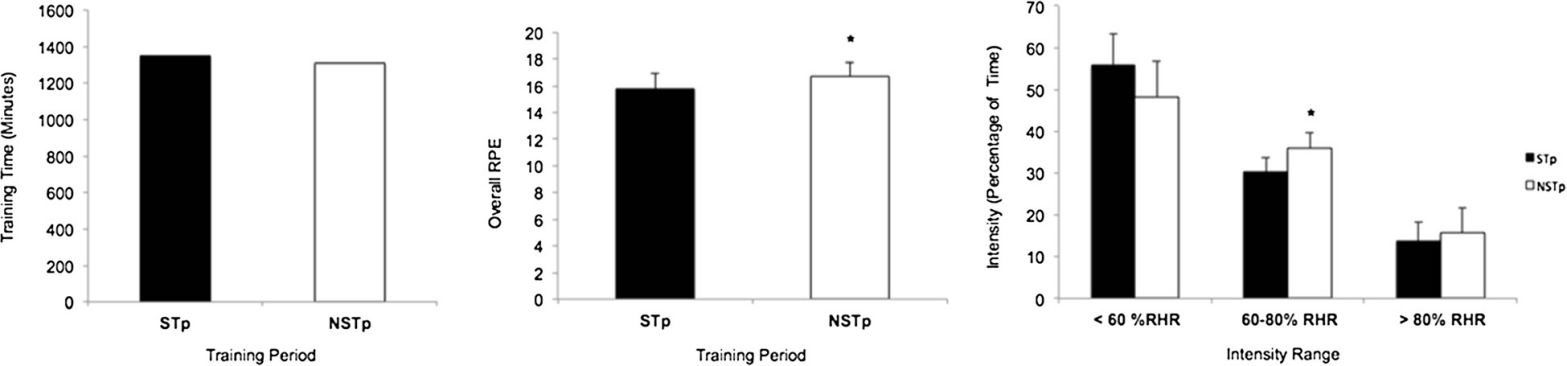
Comparison of training variables throughout the experimental trial.^*^Statistically significant difference (P < 0.05) STp vs NSTp.

The results in Figure 1 show the training loads recorded during the study period. Training load is reported here as training time, RPE and distribution among three levels of intensity during the intervention (STp) and post-intervention periods (NSTp). There were no statistically significant differences in training time between STp and NSTp.

Overall RPE during STp was significantly lower (*P* < 0.05) than during NSTp. With regard to the durations of different RHR levels (training intensity), a significant difference (*P* < 0.05) was found for the 60%–80% range, which accounted for 30.35% of the total training time during STp, and for 35.87% of the training time during the NSTp. There were no significant differences for training intensity levels in the <60% range or the >80% range.

Bivariate analysis to calculate Pearson’s correlation coefficient detected statistically significant correlations (*P* < 0.01) between overall RPE and training intensity levels of 60%–80% RHR (*r* = 0.64) and >80% RHR (*r* = 0.76).

### Biochemical assays

The results of biochemical analyses are shown in Table 4. There were no significant changes in plasma folic acid at any time point, and all values were within the normal range for the healthy population. However, plasma concentrations of Hcy increased significantly (*P* < 0.05) to above the normal range of values during the Week 8 and Week 16 periods compared to baseline values in Week 0. Regarding the relationship between plasma concentrations of Hcy and folic acid and training intensity, we found that both plasma concentrations showed a significant negative correlation (*r* = −0.75) (*P* < 0.01) with the level of intensity of <60% RHR. Bivariate analysis disclosed a significant negative correlation (*P* < 0.01) between Hcy and folic acid concentrations (*r* = −0.84) in Week 8.

**Table 4:** Biochemical values of clinical and nutritional parameters at three time points

**N = 14**		**Study period**					
**Biochemical parameters**	**Reference value**	**Week 0**		**Week 8**		**Week 16**	
		**Mean**	**SD**	**Mean**	**SD**	**Mean**	**SD**
Transferrin (mg/dl)	200 – 360	261.21	27.82	261.71	33.00	265.50	28.67
Prealbumin (mg/dl)	20 – 40	26.76	3.53	27.19	3.12	26.76	2.77
HDL (mg/dl)	40 – 60	58.29	13.58	57.29	12.28	61.00^a,b^	13.31
LDL (mg/dl)	70 – 150	74.00	22.89	71.35	20.84	83.07 ^a,b^	22.58
Total cholesterol (mg/dl)	110 – 200	147.86	26.74	149.71	27.68	154.57^a^	26.80
Folic acid (ng/ml)	4.2 – 19.9	8.14	1.17	7.73	2.57	7.62	2.36
Homocysteine (μmol/l)	5 – 12	11.64	2.65	13.92^a^	2.39	13.14^a^	1.96

HDL, high-density lipoprotein cholesterol; LDL, low-density lipoprotein cholesterol.

^a^ Statistically significant differences (*P* < 0.05) Week 0 vs. Week 8 and Week 16.

^b^ Statistically significant differences (*P* < 0.05) Week 8 vs. Week 16.

The other nutritional parameters studied here (albumin and prealbumin) showed no statistically significant changes at any time point. Among the lipid parameters we measured, HDL, LDL and total cholesterol were significantly higher (*P* < 0.05) in Week 0 compared to Week 16, and HDL and LDL were significantly higher in Week 8 compared to Week 16.

## Discussion

The results of the present study suggest that after the dietary and educational intervention, there were no significant changes in plasma concentrations of folic acid. However, we did note changes in plasma Hcy levels, despite the significant inverse correlation between the two values. Folic acid supplementation may have reduced cardiovascular risk during the NSTp in the handball players we studied.

In the present study, increased food intake as a result of nutritional education may have contributed to weight maintenance throughout the experimental period, which would avoid possible alterations in body weight as a result of poor dietary habits [1]. Regular PA is known to alter the requirements for certain micronutrients [1]. Folic acid intake in the athletes studied here (Table 2) was below the RDA except during Week 8, and was similar to the values reported by Rousseau et al. [12]. In this connection, a meta-analysis by Woolf and Manore [1] concluded that most studies which had analyzed folic acid intake based on a 3-day (72-h) recall period obtained values similar to those found in the present study. Supplementation with folic acid was implemented after an initial evaluation which showed the intake of this nutrient to be inadequate. The amount used in the dietary supplement was consistent with the theoretical basis described by McNully et al. [11], who suggested that doses of 0.2 to 0.4 mg folic acid per day may achieve maximal reductions in Hcy in healthy young people, whereas doses up to 0.8 mg folic acid per day would be needed to reduce Hcy in individuals with coronary artery disease. However, in the present study plasma Hcy concentration did not change despite the significant increase in folic acid intake.

**Figure 2:**
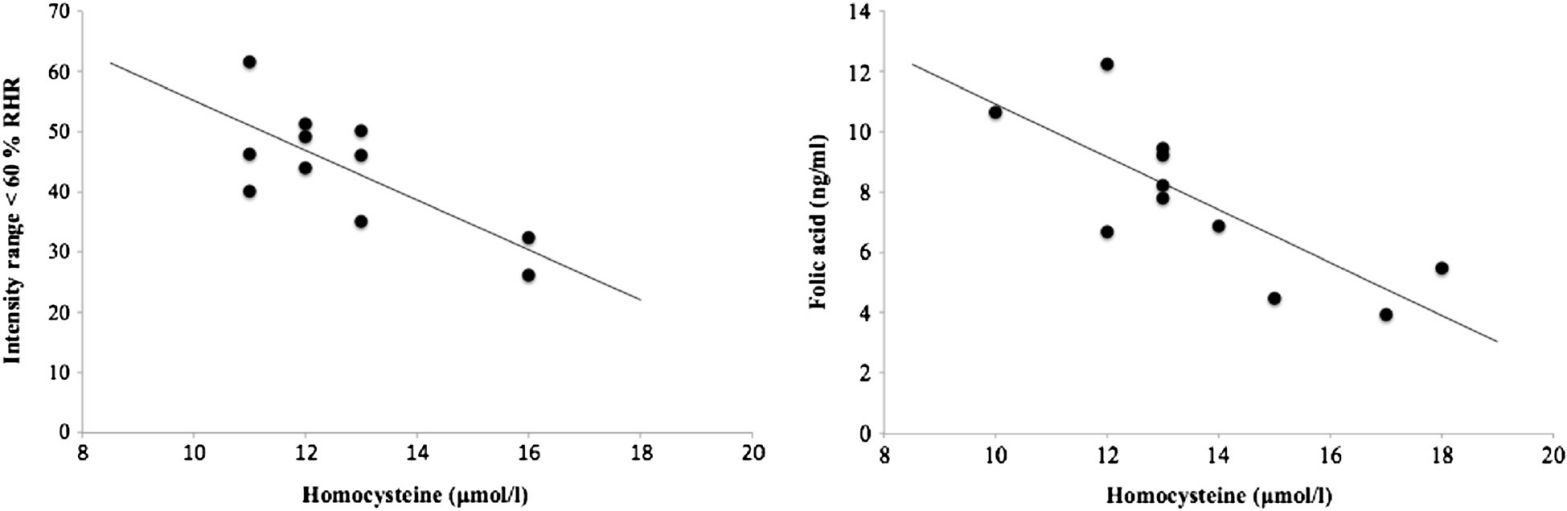
Relationship between homocysteine with other parameters in handball players.

Regular PA is known to reduce the risk of CVD [6,12]. Handball, like other team sports such as soccer and field hockey, is considered an intermittent intensity sport on the basis of the aerobic energy pathways involved [31]. When we analyzed training load, we found a significant negative correlation between exercise training time at an intensity range of <60% RHR and plasma Hcy level (Figure 2). Rousseau et al. [12] reported that athletes who performed aerobic exercise had lower levels of Hcy. This finding is consistent with our results; moreover, our direct method for quantifying training load provided data that can be considered accurate and reliable. However, a potential limitation that should be taken into account is that the present study was done under actual training conditions, although it seems that a better study design would have been to (prospectively) control the volume and intensity of PA to keep them equal among participants.

Other authors reported different values for Hcy levels after exercise; the variations among different studies may reflect the use of indirect methods to quantify PA, the lack of nutritional studies and differences between studies in mean age of the participants [4,31,32].

It is worth noting that folic acid levels in plasma were near the lower limit of normality. Other authors found that a 5-mmol/l increase in plasma Hcy levels (>10 mmol/l) was associated with a 60% increase in the risk of coronary artery disease in men [8,33]. McCully [10] noted that if the concentration of Hcy is between 8 and 12 mmol/l, improvements in the quality of the diet are needed to provide adequate vitamin intakes able to maintain Hcy at concentrations that can reduce the risk of coronary disease in adults. As described in the Results section, there was a significant negative correlation between plasma Hcy levels and plasma folic acid levels in Week 8. However, Hcy concentration increased despite dietary folic acid supplementation. This finding suggests that in contrast to the expected increase in plasma folic acid concentrations and decrease in Hcy, the opposite effect was likely attributable to training. In most participants in the present study, plasma levels of folic acid were near the lower limit of the reference values (4.2–19.l ng/ml), and after the intervention there was no significant change at the end of the supplementation period or at the end of the post-supplementation period. König et al. [5] showed that the increase in Hcy was dependent on the initial plasma level of folic acid as well as on training time. These authors attributed the increase in Hcy to increased methionine catabolism, which induced a greater influx of molecules with methyl groups as a result of high-intensity PA [4]. A study by Borrione et al. [15] analyzed team sports similar to handball but did not use dietary supplementation. They found Hcy levels that were much higher than those we found, and folic acid levels similar to those in the athletes we studied.

Our experimental approach was designed to evaluate training load, nutritional and biochemical indicators in an integrated manner to obtain accurate data in professional athletes during the sports season. Our method emphasized accurate data capture for both training load and dietary intakes. Variations in either of these factors can affect plasma levels of Hcy and folic acid, so it was important to avoid alterations that might compromise the data this study was designed to seek.

## Conclusions

Our study appears to be the first to use careful controls for participants’ training load and nutritional and biochemical status before, during and after the professional sports season. Our results suggest that high-performance athletes such as handball players may require preventive dietary supplementation with folic acid to curtail the effects of a sharp increase in blood Hcy concentrations. This increase may be associated with a sudden increase in the risk of CVD as a result of the high training load accumulated in successive training sessions during the professional competition season.

## Abbreviations

Hcy: Homocysteine; PA: Physical activity; RDA: Recommended daily allowance; RHR: Residual heart rate; RPE: Rate of perceived exertion; SD: Standard deviation.

## Competing interests

The authors declare no conflicts of interest.

## Authors’ contributions

All the authors contributed to and approved the final manuscript.
